# Predictive modeling for odor character of a chemical using machine learning combined with natural language processing

**DOI:** 10.1371/journal.pone.0198475

**Published:** 2018-06-14

**Authors:** Yuji Nozaki, Takamichi Nakamoto

**Affiliations:** Tokyo Institute of Technology, Institute of Innovative Research, Yokohama, Kanagawa, Japan; Duke University, UNITED STATES

## Abstract

Recent studies on machine learning technology have reported successful performances in some visual and auditory recognition tasks, while little has been reported in the field of olfaction. In this paper we report computational methods to predict the odor impression of a chemical from its physicochemical properties. Our predictive model utilizes nonlinear dimensionality reduction on mass spectra data and performs the clustering of descriptors by natural language processing. Sensory evaluation is widely used to measure human impressions to smell or taste by using verbal descriptors, such as “spicy” and “sweet”. However, as it requires significant amounts of time and human resources, a large-scale sensory evaluation test is difficult to perform. Our model successfully predicts a group of descriptors for a target chemical through a series of computer simulations. Although the training text data used in the language modeling is not specialized for olfaction, the experimental results show that our method is useful for analyzing sensory datasets. This is the first report to combine machine olfaction with natural language processing for odor character prediction.

## Introduction

The source of smells is airborne chemical molecules. Olfactory receptor neurons within the olfactory epithelium are activated when they bind with molecules and provide electrical signals to olfactory nerves. Then the signals are delivered to the olfactory bulb and form a pattern on it. Afterwards, on the basis of the response pattern on the olfactory bulb, comprehensive information processing associated with emotion and memory is performed in the cerebrum [1]. As each type of olfactory receptor has different molecular selectivity, the pattern of stimuli appearing on the olfactory bulb varies from molecule to molecule [2]. That is, the impression of odor also varies from molecule to molecule. From previous studies, one of the key factors contributing to differences in patterns is considered to be the molecular structure [3], [4]. If we can predict the smell impression from the physicochemical properties of a molecule, it will be an important breakthrough for the cosmetic, beverage, and food industries because a large number of experienced panelists are currently required to create the desired odors through trial and error in these industries.

Sensory evaluations using linguistic descriptors are commonly performed to quantify the odor characters of molecules. A well-known example of the sensory evaluation of odors was conducted by the research team led by Dravnieks [5]. On the basis of their evaluation tests, they proposed 146 descriptors as necessary to describe the impression of the smell of odorous substances, and they created a database of odors of 160 substances. Sigma-Aldrich has published a catalog of chemicals in which more than 1,000 monomolecular odorous substances are profiled using several hundred descriptors [6]. In the catalog, the odor character of each substance is described by the presence or absence of about 150 different descriptors (S1 and S2 Tables); therefore, a degree of odor character can not be expressed. Although the information in the catalog may sometimes be insufficient to characterize the odor of a chemical in detail, it is still a valuable tool because it describes how the chemical smells.

We previously proposed a predictive model of odor impression using a nine-layer neural network [7]. In the predictive model, which uses the results of Dravnieks’ evaluation test, 146 different odor characteristics were predicted with a correlation coefficient of 0.76 on a validation set. Essentially, the larger the sample size, the more accurately the predictive model becomes. However, with two reasons, conventional predictive modeling technique is difficult to apply; (1) the dataset contains binary values only, and is a highly sparse matrix, (2) Similar descriptors, such as 'floral' and 'rose', are exclusively used, resulting in vanishing of correlations between descriptors. In this study, we propose a mathematical model for predicting the odor category of molecules by inputting the mass spectrum, which is one of the physicochemical parameters of a molecule, after clustering according to the similarity of verbal descriptors calculated by natural language processing.

## Data

Mass spectra are physicochemical properties representing structural information of molecules and are given as a plot of intensity vs m/z (mass-to-charge ratio). The mass spectrum is uniquely determined for each molecule given the same measurement conditions. Large-scale mass spectrum datasets are available as it is possible to perform a number of mass spectrum measurements under a uniform condition.

The mass spectrum dataset used in this study was obtained from the Chemistry Webbook provided by National Institute of Standards and Technology [8]. The database consists of more than 100,000 chemicals of mass spectra acquired with electron ionization of 70[eV]. Intensities at m/z below 50 generally originate from odorless molecules such as oxygen, nitrogen, and carbon dioxide, and intensities at high m/z originate from molecules with low volatility and little effect on odor characteristics. Therefore, intensities between 51 and 262 m/z were extracted from the original dataset for this study. Then the elements in the extracted dataset were normalized by dividing by the maximum value in the dataset to obtain values between 0 and 1.

Taking the chemicals listed in both the Sigma-Aldrich catalog and NIST’s dataset, we obtained 999 samples for our experiments. We chose 138 descriptors out of 150 from the dataset since the other 12 descriptors only appeared three times or less in the catalog. As a result, the sensory data and the mass spectrum data form a matrix of 138 x 999 and a matrix of 251 x 999, respectively. In the following section, we will consider a model that connects these two datasets.

In this paper, we propose an odor character predictive model constructed from the “*Flavors and Fragrances*” catalog published from Sigma-Aldrich. As mentioned earlier, the applicability of the descriptor is given in a binary form in the Sigma-Aldrich catalog, whereas it is given on a scale of 0 to 5 in Dravnieks’ sensory evaluation. For such binary representation data, however, the number of samples in a database is much larger than that in the detailed sensory test typified by Dravnieks’ sensory evaluation, indicating the possibility of proposing a more versatile predictive model.

## Odor character predictive model

We propose a neural network that predicts the presence or absence of a specific descriptor from the mass spectrum of a chemical molecule. The input units of the neural network correspond to the m/z values of the mass spectrum, and the output units correspond to the descriptors of the odor impression.

This neural network can be trained by supervised learning with the backpropagation algorithm [9]. First, a vector representing the mass spectrum of a chemical is fed into the neural network as the input. A vector representing the odor character of the corresponding molecule is then given to the network as the desired output. Since only the presence or absence of odor character is described in the catalog, the state of the output should be 0 or 1. The error between the output of the neural network and the desired output is calculated and then network parameters (weights and biases) are modified to reduce the error.

However, a predictive model trained with the above conventional training method did not show promising performance on a validation set. One possible reason for the failure of training is that the correlations among descriptors, which should be maintained, are lost in the catalog. For example, although it can be easily imagined that "Balsam" and "Balsamic" describe a similar impression, the correlation coefficient between the two descriptors was less than 0.01 in the catalog. One hypothesis that explains this phenomenon is that only the most applicable descriptors are selected mutually exclusively from descriptors with similar impressions. For example, the three descriptors, "Rose", "Violet", and "Lavender", which are considered to represent relatively similar characters, share small correlation coefficients in the catalog dataset (Table 1) while they share larger correlation coefficients in Dravnieks’ sensory evaluation tests (Table 2).

**Table 1:** Correlation coefficients between three descriptors (catalog).

	Rose	Violet	Lavender
Rose	1	0.02	0.08
Violet	0.02	1	0.00
Lavender	0.08	0.00	1

**Table 2:** Correlation coefficients between three descriptors (Dravnieks).

	Rose	Violet	Lavender
Rose	1	0.95	0.79
Violet	0.95	1	0.84
Lavender	0.79	0.84	1

Therefore, we consider that low correlation coefficients between descriptors with similar odor characters may make it difficult for a neural network to learn vector representations of odor characters.

## Proposed model

In this paper, we propose an approach to predicting odor characters of chemicals using clusters with larger granularity containing similar descriptors. For example, all similar descriptors such as “Rose”, “Violet”, and “Lavender” are grouped in the same cluster. This cluster may represent applicability to "flower".

Aroma wheels or fragrance wheels are traditional tools for classifying odor character descriptors [10]. These tools are made by panelists with many years of experience and are commonly used to classify odors such as food, agricultural products, cosmetics and perfumes. However, the numbers of descriptors used in general aroma wheels and fragrance wheels are at most tens. Since the number of descriptors used in this study is much larger, a data-driven classification method, such as nonparametric hierarchical clustering is necessary.

To obtain a better predictive model, a hierarchical clustering method using a dendrogram was applied to the dataset. A dendrogram helps to determine the level or scale of clustering that is most appropriate for an application. Two types of scale are compared in this study. One is a similarity matrix obtained by calculating the correlation coefficient between all possible vector pairs.

The other is the similarity of words, calculated by the natural language modeling method called word2cec. Word2vec is a language modeling method proposed by Mikolov et al. at Google. Inc [11], [12]. The model uses a three-layer neural network called a skip-gram model trained with a large text corpus and is used to obtain a vector representation of words. The words included in the training corpus are projected to the word vector space as corresponding vectors. In the Word2vec training scheme, the more words share a common context in the training corpus, the shorter the distance between them in the word vector space. This enables a word vector representation to be acquired with a high probability of having such words together.

In this study, the similarity between words was calculated as the cosine distance between word vectors by Word2vec, and clustering was performed using a tree diagram based on the distance. In this paper, the full text of English Wikipedia (enwiki-201509001, 12.4GByte [13]) corpus was used with the terms of service as a training corpus. The hyperparameters of the skip-gram model are shown in Table 3.

**Table 3:** Hyperparameters used in the skip-gram model.

Hyperparameter	Value
# Units in hidden layer	400
Window size	10
Minimum count	5

The window size determines the maximum distance between words within a sentence.

All words with total frequency lower than the minimum count are ignored.

**Fig 1:**
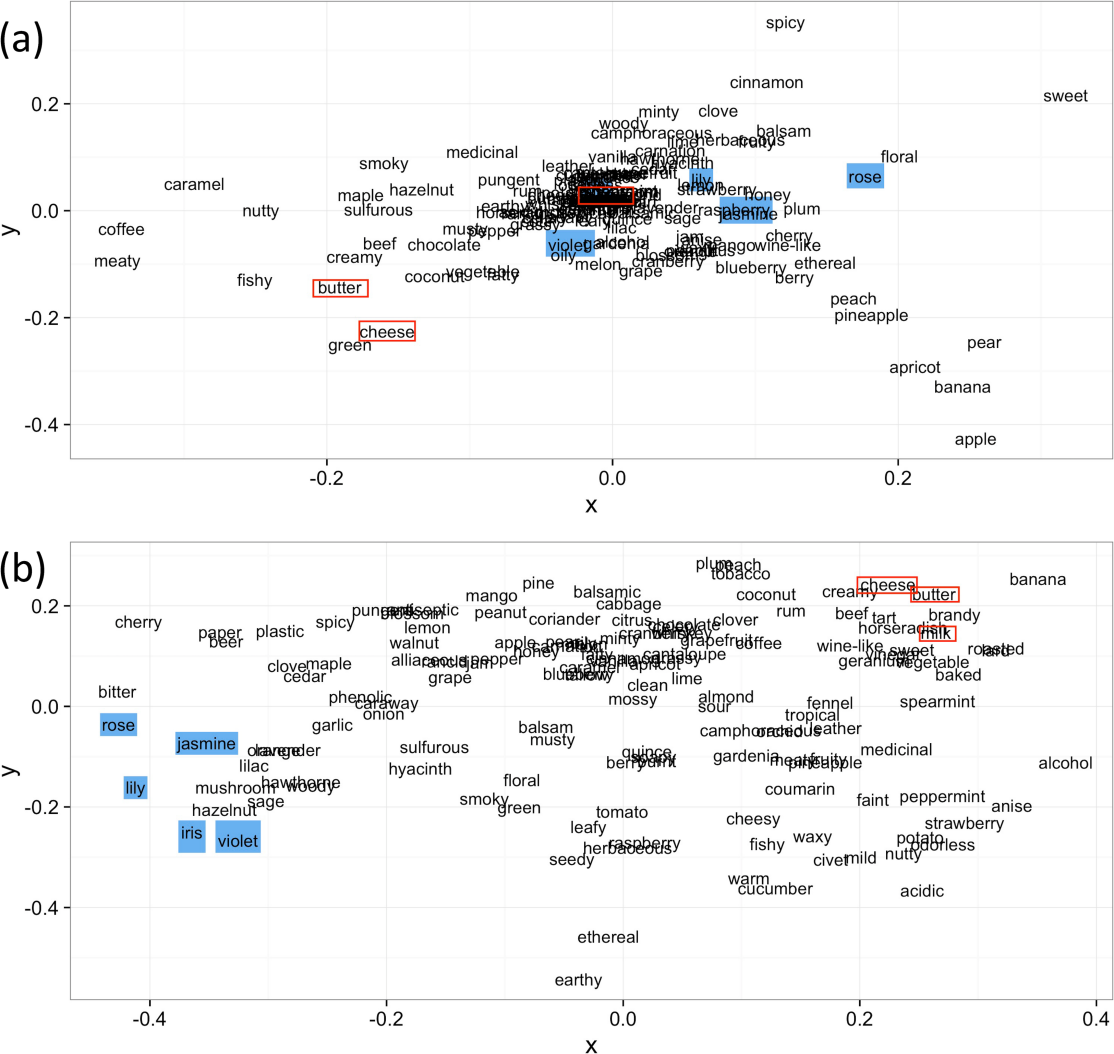
MDS diagrams based on (a) one minus the sample correlation between points, (b) cosine distance based on Word2vec modeling. *Descriptors considered belonging to the same group*, *e*.*g*. *“rose”*, *“jasmine”*, *“lily”*, *“iris” and “violet” are marked in blue*. *“milk”*, *“cheese”*, *and “butter” are enclosed in red*.

First, we visualized the positional relationship of the descriptors in two-dimensional space by multidimensional scaling (MDS) [14]. MDS visualizes the distance between points from distance or dissimilarity metrics and can produce a representation of data in a small number of dimensions. MDS requires a matrix of pairwise distances or dissimilarities. Fig 1(A) and 1(B) show the results of applying metric MDS to two matrices: the dissimilarity matrix created by subtracting the correlation coefficient between descriptors from a unit distance and the distance matrix created from the cosine distance between word vectors of the descriptors obtained by Word2vec.

**Fig 2:**
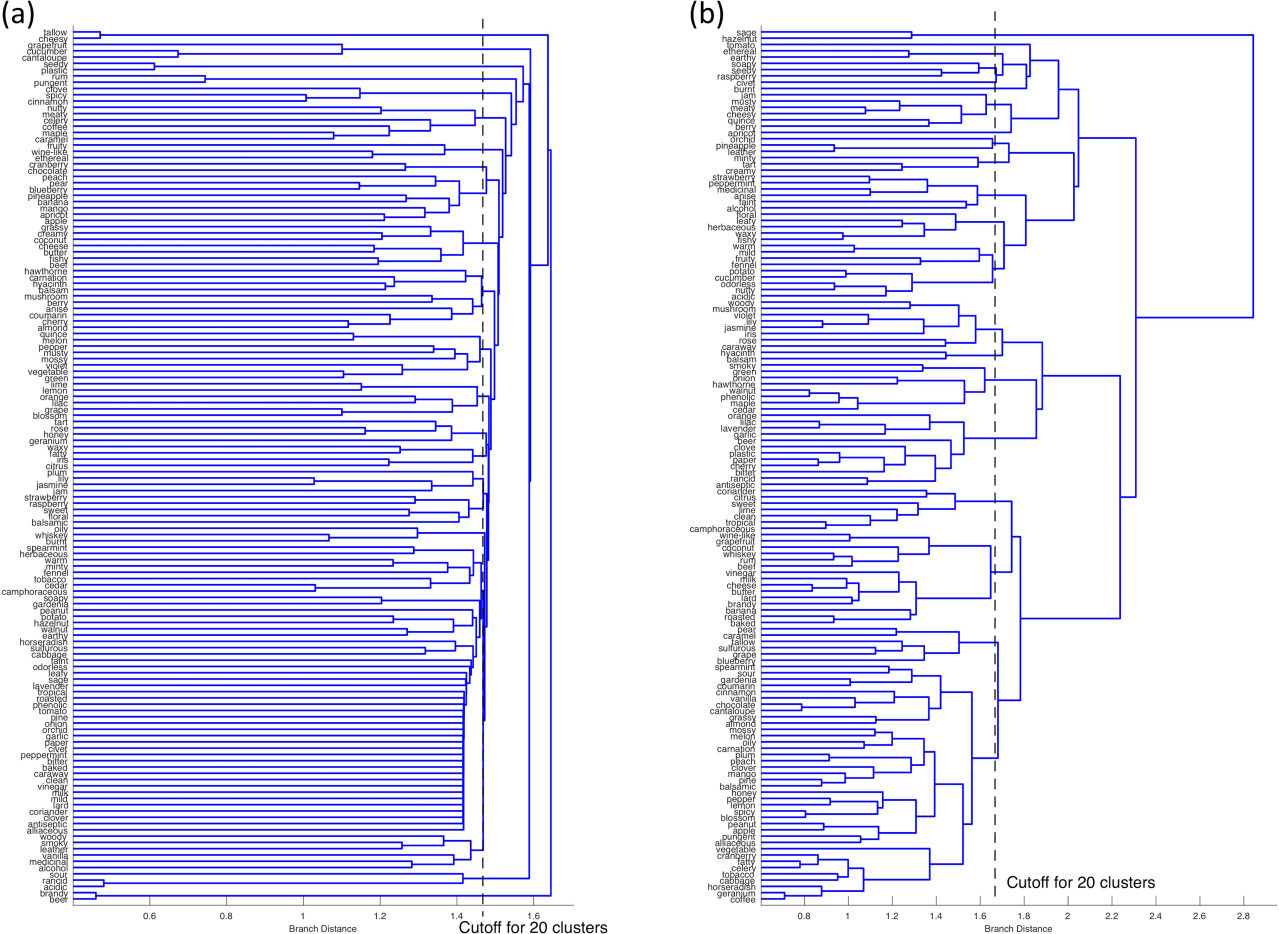
Dendrograms (unweighted average distance method) based on, (a) one minus the sample correlation between points, (b) cosine distance based on Word2vec modeling. *The dashed line in each figure shows the cut off point for twenty clusters*.

It was confirmed that the calculated similarities between the descriptors were greatly different between the two methods. As shown in Fig 1(B), in the two-dimensional diagram obtained from Word2Vec, descriptors such as “rose”, “jasmine”, “lily”, “iris”, and “violet”, and descriptors such as “milk”, “cheese”, and “butter” are placed closer than in the diagram based on the dissimilarity matrix calculated from the correlation coefficient (Fig 1(A)). Thus, we can expect that groups based on the impression of "flower" or "animal" will be formed in the two-dimensional scattering diagram obtained from Word2Vec. Dendrogram clustering based on the unweighted average distance method was performed on both the correlation coefficient matrix and the cosine similarity matrix [15]. The shape of the dendrogram is uniquely determined when the matrix of dissimilarity is given. Fig 2(A) and 2(B) show the dendrograms created from the correlation coefficient matrix and from the cosine distance matrix, respectively.

A hierarchical structure clearly appeared in the dendrogram obtained from the cosine similarity matrix, while the hierarchical structure was unclear in the dendrogram obtained from the correlation coefficient matrix. The horizontal axis of the dendrograms represents the similarity between descriptors. Therefore, the number of clusters to be separated and each descriptor contained in the cluster are determined at a cutoff point. For example, for the cutoff line shown in Fig 2(A) and 2(B), the number of clusters is twenty.

## Predictive model with clustering

**Fig 3:**
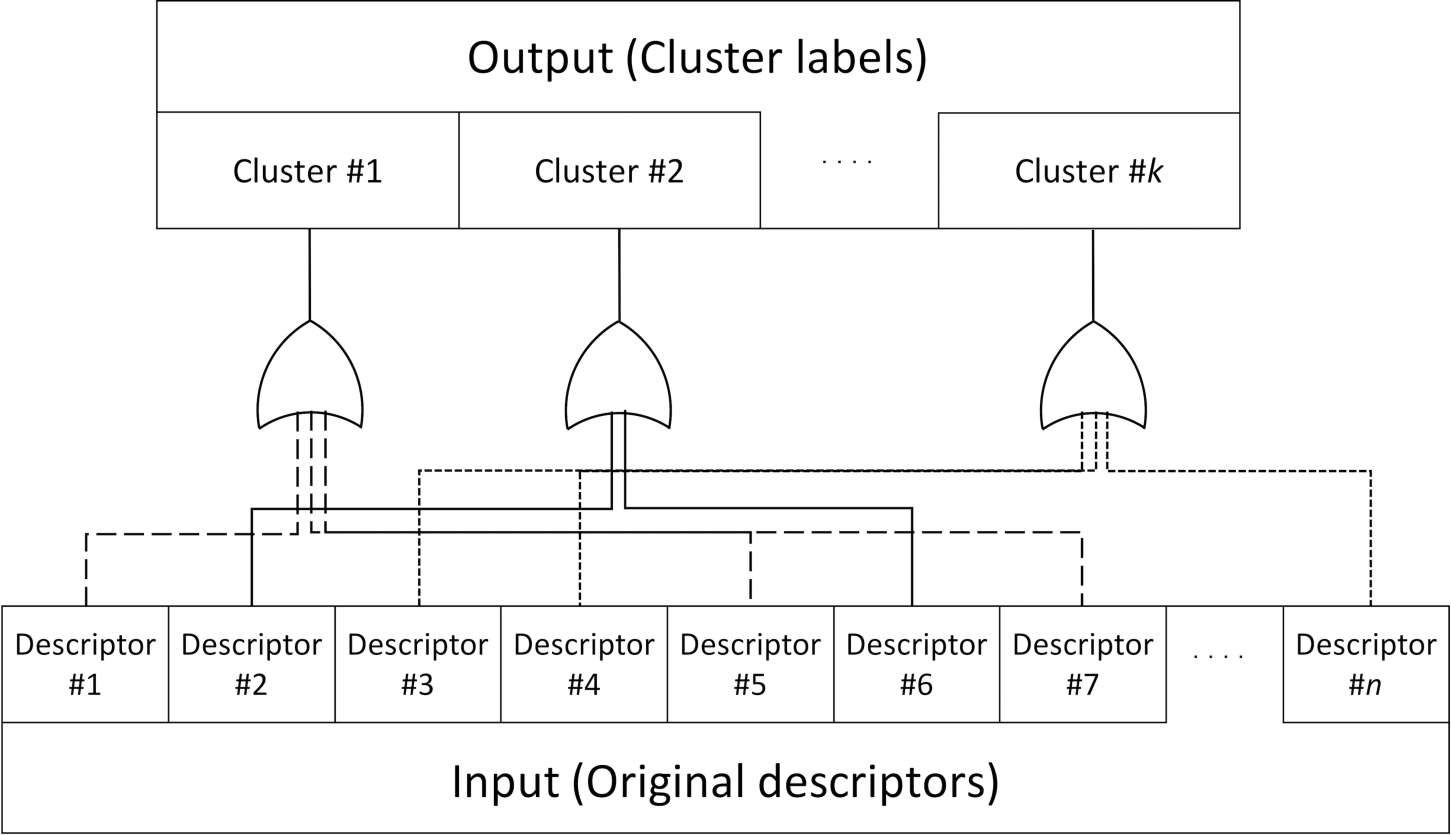
Odor character clustering. When any of the descriptors in a cluster has a value of 1, the value of the corresponding cluster that includes the sample is set to 1, e.g., if Descriptor #5 has a value of 1, Cluster #1 is set to 1.

When any of the descriptors in a cluster has a value of 1, the value of the corresponding cluster that includes the sample is set to 1. For example, when "rose", "lavender", and "iris" belong to a certain cluster, a sample having at least one of the three descriptors in the original catalog data is regarded as having the odor character of the cluster. (see Fig 3).

**Fig 4:**
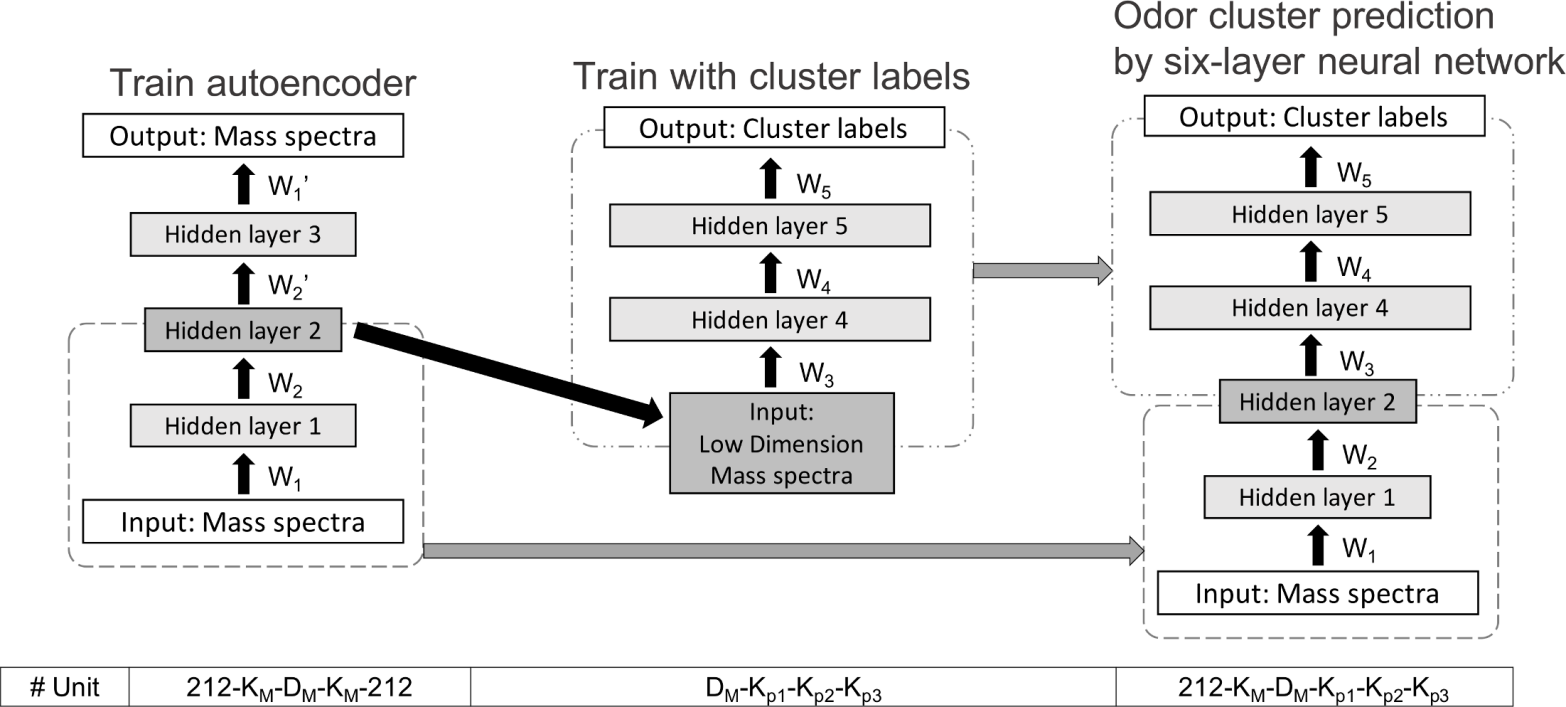
Odor character predictive model.

The predictive model proposed in this study is a six-layer neural network (Fig 4). A mass spectrum is given as the input, and a vector indicating the presence or absence of odor clusters is given as the desired output. As mass spectra are high-dimensional, an autoencoder is used for dimensionality reduction [16]. The size of the feature vector is set to 30 through an optimization process, where we evaluate the residual between the input and output by cross-validation. Because of the problem known as "the curse of dimensionality", the autoencoder’s performance degrades when the size of the feature vector is not optimized [17]. The autoencoder used in this study consists of five layers including three hidden layers, as shown in Fig 4. After the original mass spectra of 212 dimensions are converted into feature vectors of 30 dimensions, each feature vector is fed to the four-layer neural network which gives odor cluster vector as the desired output.

To compare the generalization performance of the model, five-fold cross validation was performed on the dataset. Since 999 is indivisible by 5, the numbers of samples included in the five subsets were [200, 200, 200, 200, 199].

The performances of the predictive models were evaluated in terms of accuracy. Let the input be ***x***, the weights and biases of the predictive model be ***w*** and ***b***, the model’s output vector be *f*(***x***;***w***,***b***) and the desired output be ***y***. Then, the accuracy of the predictive model is calculated as the rate of concordance between the model’s output and desired output.

First, the autoencoder, shown in the left part of Fig 4, is a special type of neural network that is trained to reproduce an input. It has a bottleneck in the middle hidden layer and is thus also called a sandglass-style neural network. As the name implies, the number of inputs is equal to the number of outputs. As the autoencoder has fewer hidden nodes in the middle layer than nodes in the input or output layer, this structure compresses data into a smaller dimension (feature vector). Then the mapping neural network shown in the middle part of Fig 4 is trained to output cluster labels from a feature vector. Finally, these two neural networks are connected to form a neural network that predicts the label of the cluster from a mass spectrum (Fig 4 right). The final output of the neural network is converted to 0 or 1 by a step function. The threshold of the step function used in each model is optimized to maximize the performance for the training set. A sigmoid function was used as the activation function in the hidden layer and output layer of both the autoencoder and the mapping neural network.

The mean squared error was used as the error function of the neural networks. Training was carried out by error backpropagation using stochastic gradient descent [18].

Weights and biases were updated using the following function; where ***w***^(τ)^ is the weight vector at the τth epoch and *E*(***w***^(τ)^) is the error function,

$$w^{\left(\tau +1\right)}=w^{\left(\tau \right)}-\eta \frac{\partial E(w^{\left(\tau \right)}))}{\partial w^{\left(\tau \right)}}+\alpha (w^{\left(\tau \right)}-w^{\left(\tau -1\right)})$$ (1)

Here, learning rate *η*, is a positive constant used to control the learning speed and α is a positive constant applied the momentum term to accelerate convergence [19].

To avoid overfitting of the model, a regularization term was added to the error function as shown in Eq (2) [20], [21]. As the complexity of the model increases, the regularization term acts as a penalty. Let *E*_*n*_(***w***) be the error at the *n*th neuron in the output layer, *N* be the number of neurons in the output layer, and ***W*** be all the weights in the predictive model. Then

$$E(w)=\frac{1}{N}\sum_{n}^{N}\;E_{n}(w)+\lambda {\left\|W\right\|}^{p}$$ (2)

where *λ* is a positive constant used to determine the contribution of the regularization term and p is the norm of the regularization term. All the weights were initialized to have a Gaussian distribution with zero mean and a standard deviation of 0.1.

The hyper parameters of the predictive model, namely, the number of clusters, the number of neurons in each layer, the learning rate *η*, the momentum constant α, and the regularization constant *λ*, were optimized in a series of simulations and are summarized in Tables 4 and 5. Since the predictive model includes two neural networks (the autoencoder and the mapping neural network), hyperparameters for the autoencoder are displayed with a subscript of M, whereas the hyperparameters for the mapping neural network are displayed with subscript P. The number of output clusters *K*_*p*3_ is determined on the basis of experimental results.

**Table 4:** Hyperparameters in predictive model (Word2vec-based).

Hyperparameter	Value
*K*_*M*_	85
*D*_*M*_	30
*K*_*p*1_	50
*K*_*p*2_	20
*K*_*p*3_	-
η_*M*_	0.5×0.99^*τ*^
η_*p*_	0.3×0.99^*τ*^
α_*M*_	0.3×0.99^*τ*^
α_*p*_	0.1 × 0.99^*τ*^
λ_*M*_, p	3 × 10^−7^,L2
λ_*p*_, p	2 × 10^−7^,L2
# epochs in MS autoencoder training	1,000,000
# epochs in mapping network training	500,000
# epochs in fine tuning	1,000

**Table 5:** Hyperparameters in predictive model (correlation-based).

Hyperparameter	Value
*K*_*M*_	85
*D*_*M*_	30
*K*_*p*1_	50
*K*_*p*2_	20
*K*_*p*3_	-
η_*M*_	0.5×0.99^*τ*^
η_*p*_	0.3×0.99^*τ*^
α_*M*_	0.3×0.99^*τ*^
α_*p*_	0.1 × 0.99^*τ*^
λ_*M*_, p	3 × 10^−7^,L2
λ_*p*_, p	2 × 10^−7^,L2
# epochs in MS autoencoder training	1,000,000
# epochs in mapping network training	1,000,000
# epochs in fine tuning	1,000

## Results of model performance

**Fig 5:**
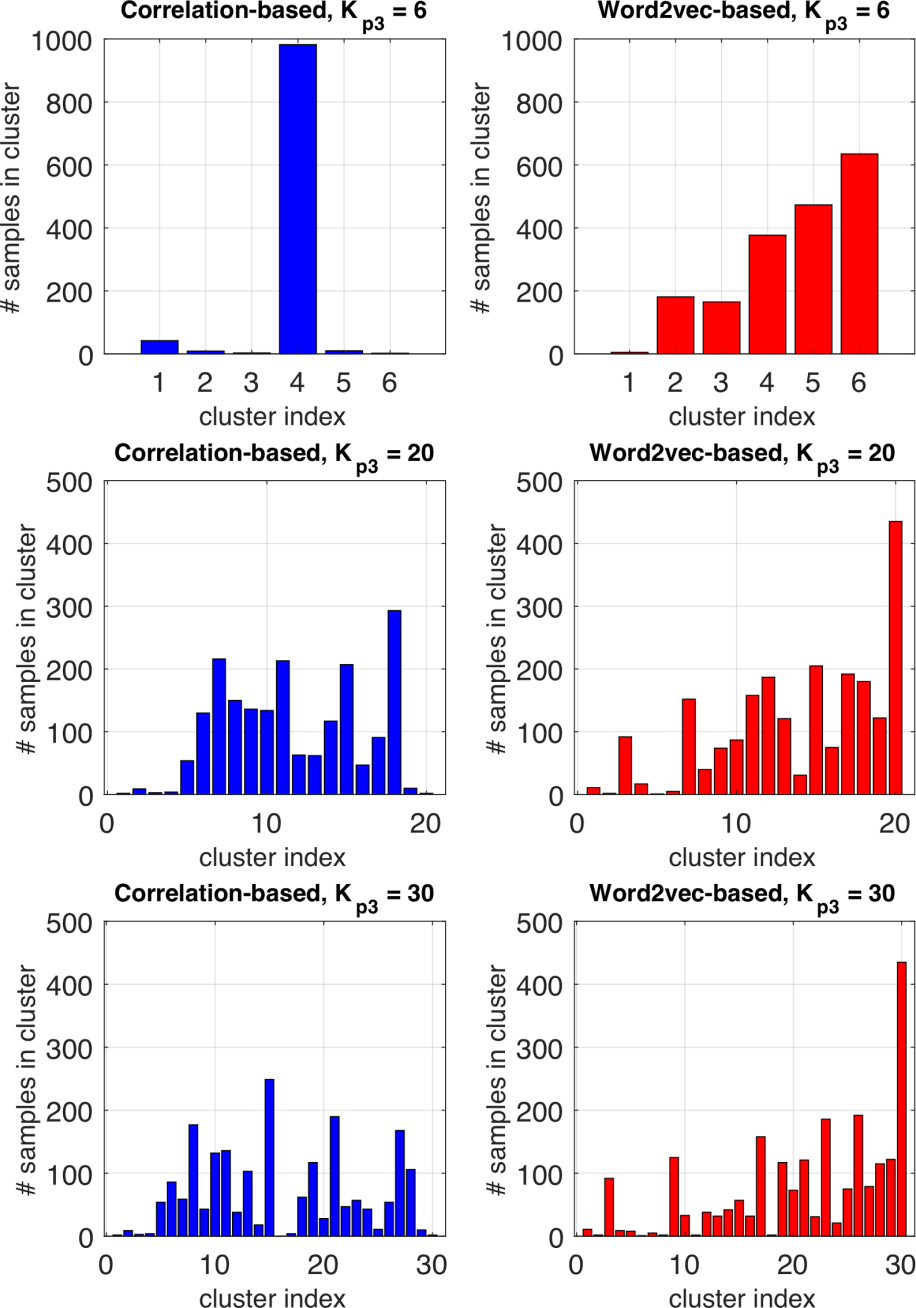
Distribution of samples with respect to *K*_*p*3_.

The number of cluster *K*_*p*3_ must be determined carefully since the distribution of samples strongly affect performance of the predictive model. Fig 5 shows the distribution of samples with respect to *K*_*p*3_. As shown in Fig 5, sample distribution of correlation-based model is out of balance from cluster to cluster when *K*_*p*3_ is 6. This means that most of the descriptors belong to one huge cluster while the other clusters include very small number of descriptors. In such case, a predictive model may mark very high accuracy while the prediction provides little information.

**Fig 6:**
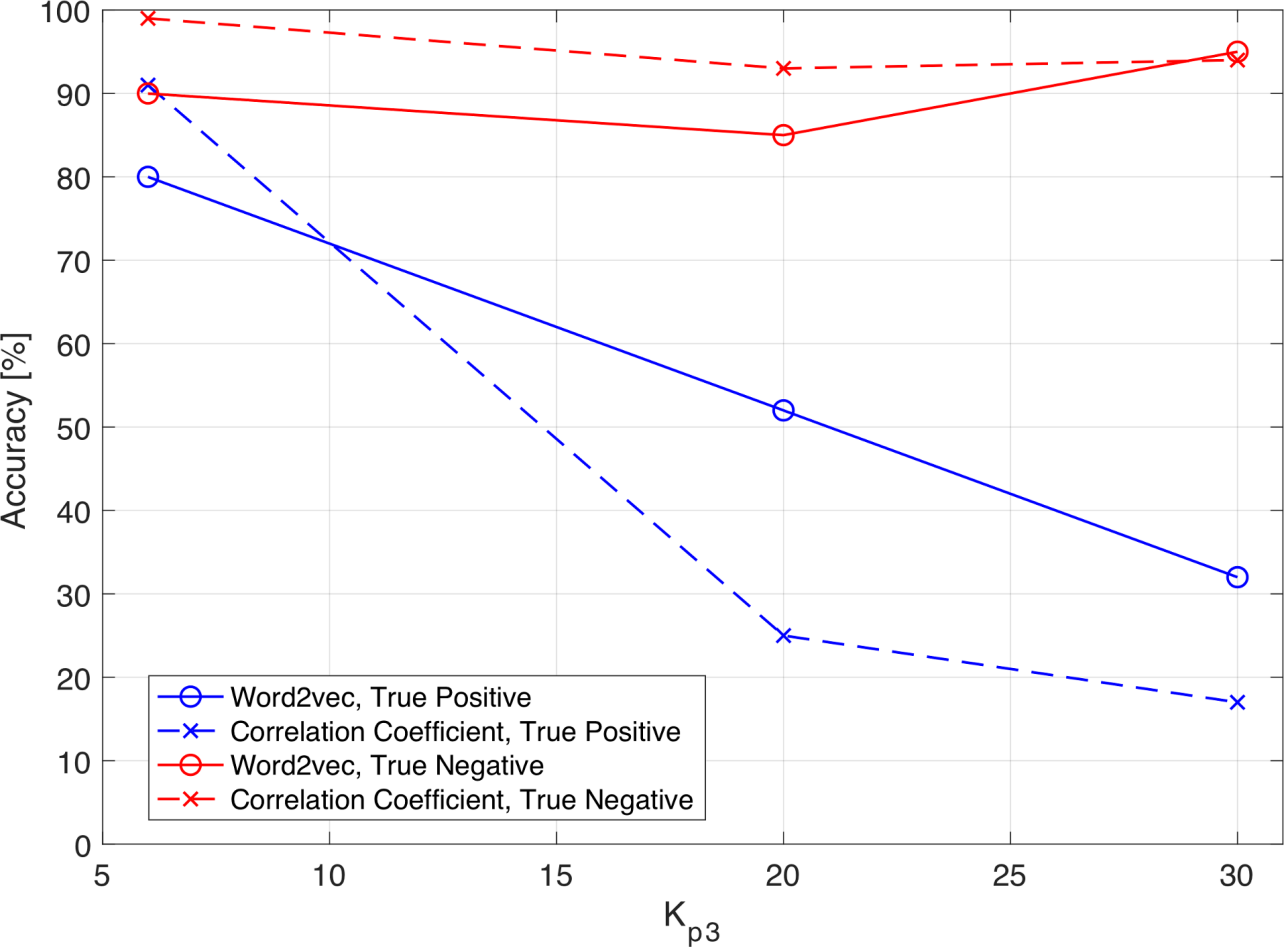
Accuracy of predictive models with respect to *K*_*p*3_.

Fig 6 shows the accuracy of the predictive models (Word2vec- and correlation-based) as a function of *K*_*p*3_. In Fig 6, the number of clusters *K*_*p*3_ in the output layer is plotted on the abscissa and the true positive or true negative value is plotted on the ordinate, where “true positive” indicates the rate at which the model outputs 1 when the desired output is 1, and “true negative” indicates the rate at which the model outputs 0 when the desired output is 0. As more than 98.4% of the sensory dataset consists of zeros, the predictive models tend to output “0”, resulting in lower accuracy of true positives.

From the results of this experiment, *K*_*p*3_ is set to 20 to ensure classification accuracy. The accuracy of the two predictive models when the number of clusters *K*_*p*3_ is 20 is shown in Tables 6 and 7. Although the number of clusters has a trade-off relationship with the accuracy of the model, in the model using clusters based on language processing, the prediction accuracy of true positives is 53% and the prediction accuracy of true negatives is 85% when the number of clusters is 20, while the model with correlation-coefficient-based clusters shows lower accuracy.

**Table 6:** Accuracy of predictive model (Word2vec).

		Desired output	
		1	0
Output	1	53 [%]	15 [%]
	0	47 [%]	85 [%]

**Table 7:** Accuracy of predictive model (correlation coefficient).

		Desired output	
		1	0
Output	1	25 [%]	6 [%]
	0	75 [%]	94 [%]

## Conclusion

In this paper, we proposed a predictive model incorporating the language modeling method Word2vec to predict odor characters of chemicals represented by binary values from mass spectra. In the catalog data of Sigma-Aldrich used in this study, descriptors to represent the odor characters of molecules are used exclusively even if other descriptors represent similar odor characters, resulting in banishing of similarity between descriptors.

Although the number of clusters is a trade-off relationship with the accuracy of the model, our proposed model had a prediction accuracy of 53% for true positives and of 85% for true negatives when the number of odor clusters was set to twenty. As shown in Table 6, the proposed model can predict odor characteristics of unknown chemicals with large granularity.

Note that Wikipedia’s text corpus used in this experiment is not particularly focused on the impression of odor. Thus, we expect that the performance of the proposed modeling method can be improved by using a dataset more devoted to smell.

## Supporting information

S1 Table List of verbal descriptors. (CSV)

S2 Table Odor character profile of chemicals. (CSV)

## References

[1] N. Takamichi, *Essentials of Machine Olfaction and Taste*, vol. 1. Wiley, 2016.

[2] Buck L. and Axel R., “A novel multigene family may encode odorant receptors: a molecular basis for odor recognition,” *Cell*, vol. 65, no. 1, pp. 175–187, 4 1991 1840504 10.1016/0092-8674(91)90418-x

[3] Araneda R. C., Kini A. D., and Firestein S., “The molecular receptive range of an odorant receptor,” *Nat*. *Neurosci*., vol. 3, no. 12, pp. 1248–1255, 12 2000 doi: 10.1038/81774 11100145 10.1038/81774

[4] Kajiya K., Inaki K., Tanaka M., Haga T., Kataoka H., and Touhara K., “Molecular Bases of Odor Discrimination: Reconstitution of Olfactory Receptors that Recognize Overlapping Sets of Odorants,” *J*. *Neurosci*., vol. 21, no. 16, pp. 6018–6025, 8 2001 11487625 10.1523/JNEUROSCI.21-16-06018.2001 PMC6763140

[5] A. Dravnieks, “Atlas of odor character profiles,” 1992.

[6] SIgma-Aldrich, “Flavors and Fragrances.” [Online]. Available: http://go.sigmaaldrich.com/ff-catalog-download-safcglobal. [Accessed: 15-Aug-2017].

[7] Nozaki Y. and Nakamoto T., “Odor Impression Prediction from Mass Spectra,” *PLOS ONE*, vol. 11, no. 6, p. e0157030, 6 2016 doi: 10.1371/journal.pone.0157030 27326765 10.1371/journal.pone.0157030 PMC4915715

[8] “NIST Chemistry WebBook.” [Online]. Available: http://webbook.nist.gov/chemistry/. [Accessed: 10-Aug-2017].

[9] Rumelhart D. E., Hinton G. E., and Williams R. J., “Learning representations by back-propagating errors,” *Nature*, vol. 323, no. 6088, pp. 533–536, 10 1986.

[10] Suffet I. H., Brady B. M., Bartels J. H. M., Burlingame G., Mallevialle J., and Yohe T., “Development of the Flavor Profile Analysis Method into a Standard Method for Sensory Analysis of Water,” *Water Sci*. *Technol*., vol. 20, no. 8–9, pp. 1–9, 8 1988.

[11] Mikolov T., Chen K., Corrado G., and Dean J., “Efficient Estimation of Word Representations in Vector Space,” *ArXiv13013781 Cs*, 1 2013.

[12] Mikolov T., Sutskever I., Chen K., Corrado G., and Dean J., “Distributed Representations of Words and Phrases and their Compositionality,” *ArXiv13104546 Cs Stat*, 10 2013.

[13] “The Wikipedia Corpus.” [Online]. Available: https://corpus.byu.edu/wiki/. [Accessed: 27-Dec-2017].

[14] Borg I. and Groenen P., “Modern Multidimensional Scaling: Theory and Applications,” *J*. *Educ*. *Meas*., vol. 40, no. 3, pp. 277–280, 9 2003.

[15] Szekely G. J. and Rizzo M. L., “Hierarchical Clustering via Joint Between-Within Distances: Extending Ward’s Minimum Variance Method,” *J*. *Classif*., vol. 22, no. 2, pp. 151–183, 2005.

[16] Hinton G. E. and Salakhutdinov R. R., “Reducing the Dimensionality of Data with Neural Networks,” *Science*, vol. 313, no. 5786, pp. 504–507, 7 2006 doi: 10.1126/science.1127647 16873662 10.1126/science.1127647

[17] Trunk G. V., “A Problem of Dimensionality: A Simple Example,” *IEEE Trans*. *Pattern Anal*. *Mach*. *Intell*., vol. PAMI-1, no. 3, pp. 306–307, 7 1979. 10.1109/tpami.1979.4766926 21868861

[18] Lecun Y., Boser B., Denker J.S., Henderson D., Howard R.E., Hubbard W. et al, “Handwritten digit recognition with a back-propagation network,” 1990.

[19] Wiegerinck W., Komoda A., and Heskes T., “Stochastic dynamics of learning with momentum in neural networks,” *J*. *Phys*. *Math*. *Gen*., vol. 27, no. 13, p. 4425, 1994.

[20] Tibshirani R., “Regression Shrinkage and Selection via the Lasso,” *J*. *R*. *Stat*. *Soc*. *Ser*. *B Methodol*., vol. 58, no. 1, pp. 267–288, 1996.

[21] Chui C. K. and Li X., “Approximation by ridge functions and neural networks with one hidden layer,” *J*. *Approx*. *Theory*, vol. 70, no. 2, pp. 131–141, 8 1992.

