# Correction: Predictive modeling for odor character of a chemical using machine learning combined with natural language processing

**DOI:** 10.1371/journal.pone.0208962

**Published:** 2018-12-05

**Authors:** Yuji Nozaki, Takamichi Nakamoto

There are errors in S2 Table. The values in the “Applicable descriptor Number (from S1 Table)” column are incorrect and should be 1 value lower. Additionally, the sensory dataset which was used in the computer simulation in S2 Table, is not perfectly equivalent to the original data source, Sigma-Aldrich’s “Flavors and Fragrances” [2]. Therefore, S2 Table data are different from the original source. Some of the descriptors may be ignored for samples described by more than 6 descriptors. As descriptors are listed ascending in alphabet, ignored descriptors are mainly: “sweet”, “vanilla” and “wine-like”. Those descriptors are used when the number of descriptors is not more than six. Approximately 11% of samples in the dataset affected.

Please see the corrected S2 Table caption and file below.

## Supporting information

S2 Table Odor character profile of chemicals. S2 data set is different from original source.
(CSV)
